# Hierarchical looping results in extreme extensibility of silk fibre composites produced by Southern house spiders (*Kukulcania hibernalis*)

**DOI:** 10.1098/rsfs.2023.0071

**Published:** 2024-06-07

**Authors:** Daniele Liprandi, Martin Ramírez, Sascha Schlüter, Lucas Baumgart, Anna-Christin Joel, Peter Michalik, Jonas O. Wolff

**Affiliations:** ^1^ Evolutionary Biomechanics, Zoological Institute and Museum, University of Greifswald, Greifswald, Germany; ^2^ Museo Argentino de Ciencias Naturales “Bernardino Rivadavia”, Consejo Nacional de Investigaciones Científicas y Técnicas, Buenos Aires, Argentina; ^3^ Institute of Biology II, RWTH Aachen University, Aachen, Worringerweg 52074, Germany; ^4^ Institute of Organismic and Molecular Evolution, Johannes Gutenberg-University, Mainz, Germany; ^5^ Zoological Museum, Zoological Institute and Museum, University of Greifswald, Greifswald, Germany; ^6^ School of Natural Sciences, Macquarie University, Sydney, New South Wales 2109, Australia

**Keywords:** spider silk, material meta-structure, fibre composite material, fabric, Filistatidae, cribellate spider

## Abstract

Spider silk is a tough and versatile biological material combining high tensile strength and extensibility through nanocomposite structure and its nonlinear elastic behaviour. Notably, spiders rarely use single silk fibres in isolation, but instead process them into more complex composites, such as silk fibre bundles, sheets and anchorages, involving a combination of spinneret, leg and body movements. While the material properties of single silk fibres have been extensively studied, the mechanical properties of silk composites and meta-structures are poorly understood and exhibit a hereto largely untapped potential for the bio-inspired design of novel fabrics with outstanding mechanical properties. In this study, we report on the tensile mechanics of the adhesive capture threads of the Southern house spider (*Kukulcania hibernalis*), which exhibit extreme extensibility, surpassing that of the viscid capture threads of orb weavers by up to tenfold. By combining high-resolution mechanical testing, microscopy and *in silico* experiments based on a hierarchical modified version of the Fibre Bundle Model, we demonstrate that extreme extensibility is based on a hierarchical loops-on-loops structure combining linear and coiled elements. The stepwise unravelling of the loops leads to the repeated fracture of the connected linear fibres, delaying terminal failure and enhancing energy absorption. This principle could be used to achieve tailored fabrics and materials that are able to sustain high deformation without failure.

## 1. Introduction

Spiders are known to construct intricate webs with thin silk fibres possessing outstanding mechanical properties [1]. Notably, each spider is able to produce multiple types of silks, each with their own function [2]. The mechanical behaviour of each silk material emerges from the specific composite structure of the proteins (including repeated crystalline, helical and amorphous domains) forming the silks [1,3,4]. These properties have spurred interest not only in the field of biology but also in material science, inspiring applications ranging from textile and sustainable packaging solutions to biomedical materials aiding in tissue engineering and drug transport [5–7]. Past and current research on spider silk, especially in the applied sciences, focuses on silk proteins (spidroins) and fibres forming the draglines and web scaffolds, so-called major ampullate silk. However, spiders produce a multitude of different silk materials, by combining and processing different silk secretions into meta-structures, such as threads (i.e. bundles of fibres or compounds of fibres and adhesives), anchors, sheets and webs, with often complex mechanical behaviours [8–11]. One of the most intricate regular silk meta-structures is found in the so-called cribellar capture threads, produced by multiple spider lineages with different web architectures [12].

Cribellate spiders are known to incorporate nanofibres with diameters below 50 nm into their capture threads [12–15], unlike their ecribellate counterparts, many of which deploy viscid glue droplets to coat their capture threads [13]. The cribellar nanofibres are extracted from the cribellum spinning organ (a flat broad plate of merged modified spinnerets) and brushed up using a comb-like structure on the hind leg, the calamistrum. The results are puffs of hundreds of nanofibres acting like an adhesive mass, that is, attached to a carrier thread composed of straight axial lines and (usually) coiled or undulating fibres [14]. These composite cribellar threads adhere through a dry adhesion mechanism based on van der Waals forces, hygroscopic forces [16], and through the interaction with the viscous secretion covering insect prey surfaces [17,18].

**Figure 1.**
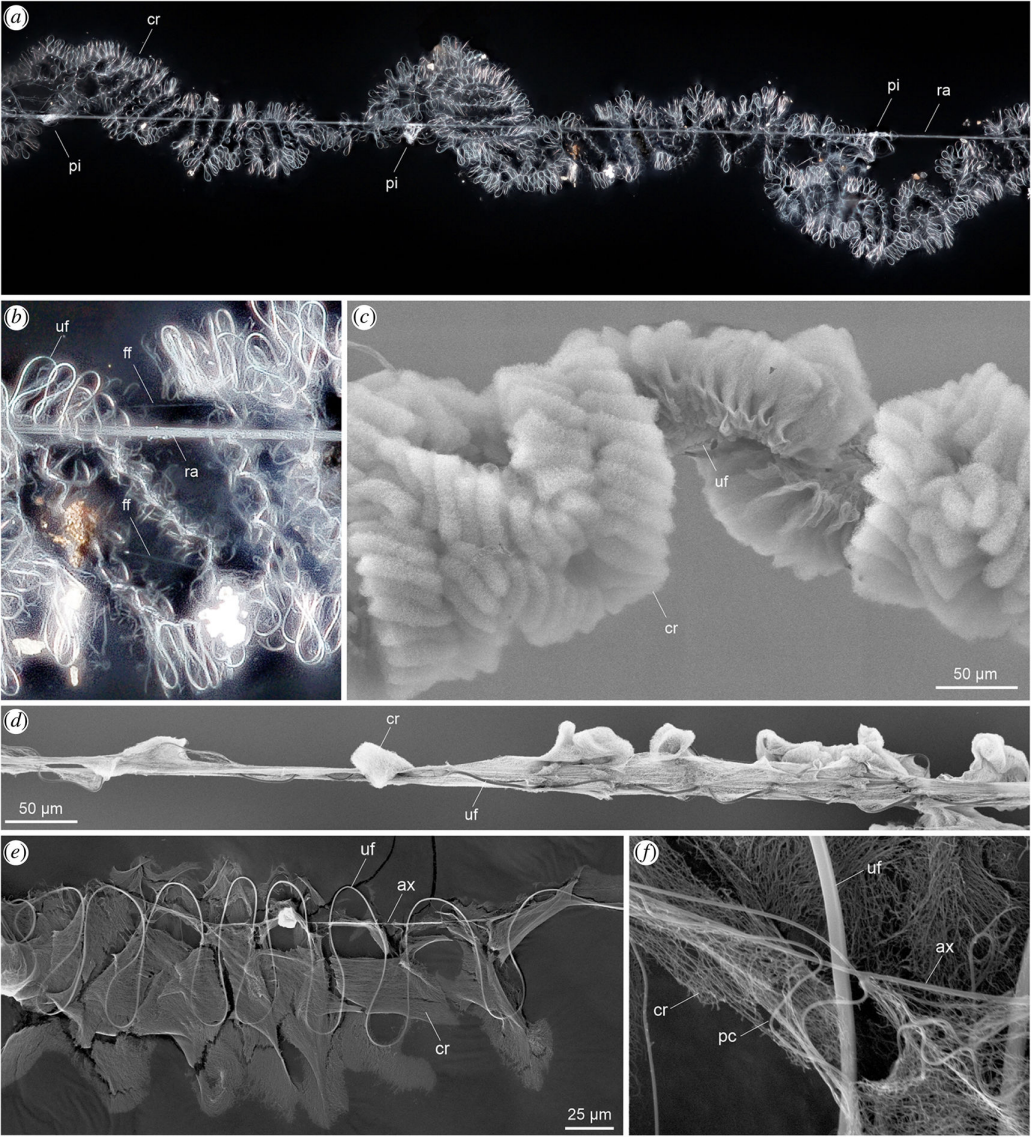
Meta-structure of the *K. hibernalis* cribellar capture threads. (*a*) Macro-photo of a capture thread segment showing the paired cribellar band coiling loosely around a straight radial line (third-order looping), to which it is tightly attached in regular intervals via piriform silk joints. (*b*) Magnified and brightened up detail from (a) showing the different fibres defining the multi-hierarchical coiling meta-structure of the thread. (*c*) Scanning electron micrograph (SEM) of the cribellar thread (without radial line). In the electron microscope, the cribellar fibre mass is well visible and covers most of the uf and pc fibres. (*d*) SEM of a partially strained cribellar thread with the cribellar mass stretched out and the uf loops partly opened and separated from the cribellar envelope. (*e*) View on the inner side of one cribellar band in non-strained state, showing the straight axial line crossing the regularly looping uf and pc fibres (first-order looping). (*f*) Detail of an intersection of the different fibres, showing their interaction. The uf and ax fibres adhere to each other in the non-strained state. ax, axial fibre; cr, cribellar fibres/mass (in (*a*) cribellar band); ff, framing fibre; pc, paracribellar fibre; pi, piriform silk joint; ra, radial line (consists of multiple fibres); uf, undulating fibre.

**Figure 2.**
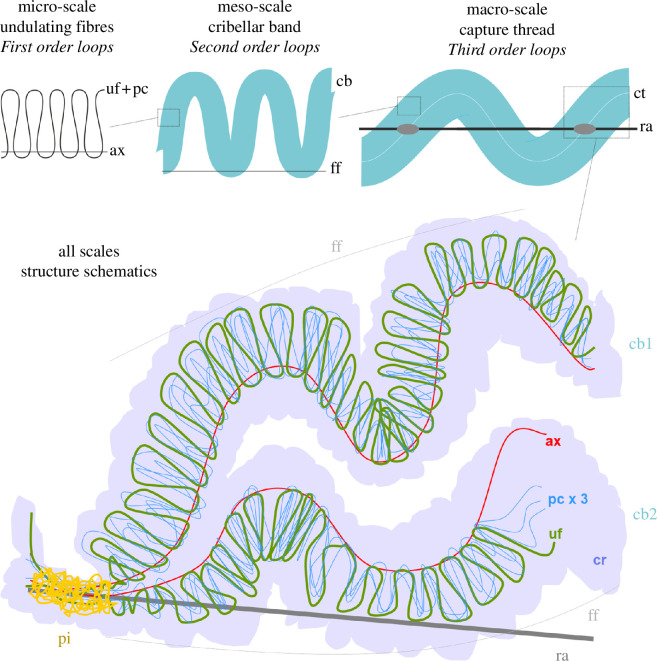
Schematic drawing of the *K. hibernalis* cribellar capture thread meta-structure. The top row displays the first- to third-order loop structure across different length scales. The drawing below illustrates the bi-hierarchical first- and second-order looping (loops-on-loops) structure in more detail, depicting the different silk fibre types included in the thread. ax, axial fibre; cb, cribellar band; cr, cribellar fibres/mass; ff, framing fibre; pc, paracribellar fibre; pi, piriform silk joint; ra, radial line (consists of multiple fibres); uf, undulating fibre.

Spider capture threads, both viscid and cribellar, are characterized by high extensibility. For example, the viscid capture threads of orb webs have been shown to extend up to 10 times their initial length before fracture [19–21]. A recent study found that the cribellar capture threads of the Australian odd-claw spider *Progradungula otwayensis* (Gradungulidae) could be extended up to more than 25 times [21]. Cribellar threads in this species exhibit undulating fibres that in themselves are tightly coiled, representing looping patterns on two-length scales that add extra extensibility by permitting high hidden fibre lengths. An even more complex structure was found in the cribellar threads in the Southern house spider *Kukulcania hibernalis* (Filistatidae), a common species distributed across the American continents. Its coiled capture threads are composed of a paired cribellar band attached to a stiff radial frame line. Each of these cribellar bands exhibits an undulating meso-structure determined by a central, so-called, axial fibre. Attached to this axial fibre are the micro-scale regularly undulating fibres of minor ampullate silk, alongside three pairs of thin looped paracribellar fibres and hundreds of cribellar nanofibres (figures 1*a*,*b* and 2) [12,13,22]. These capture threads are used by *K. hibernalis* in substrate-bound sheet webs and often laid out in a more or less radial arrangement around the tubular retreat of the spider, enabling them to capture diverse prey arthropods that walk across the substrate. It has been observed that *Kukulcania* capture threads can be pulled and extended into long strands [22], but, thus far their tensile mechanics have not been reported. It was hypothesized that due to the multi-hierarchical loop structure, the thread would exhibit extreme elongations with an expressed fracture delay, but data on the performance of this remarkable silk composite is still missing.

Looped or coiled structures have been shown to play a crucial role in high-strain biological and bio-inspired materials [23–26]. In spider silk, this principle was described both on the macro-molecular level [27,28] and on the meta-structural level. In the latter case, it is crucial that loops are held together by a force, either by the adhesion of the fibre to itself (e.g. in the looping threads of recluse spiders [8]) or to a straight fibre in the bundle (such as the axial line of cribellar threads [19]) or by capillary forces of a viscous coating such as in spooled capture threads of ecribellar orb weavers [29]. However, to date, the mechanical principle of the extreme elongation of silk meta-structures combining looped and straight fibres, as present in cribellar capture threads, is poorly understood. It is especially unclear how the hierarchically coiled (i.e. loops-on-loops) morphology, unique to *Kukulcania* threads [22], affects tensile mechanics, e.g. if coils unravel stepwise or simultaneously and how energy is transmitted between loop elements under tensile load.

In order to describe the tensile mechanics of *Kukulcania* capture threads, we combined a high-resolution tensile testing approach integrating microscopic video recording with light and field emission SEM of native and strained threads. To set the extensibility of *Kukulcania* capture threads in relation with that of a typical ecribellar orb weaver, we performed comparative vertical tension experiments, i.e. loading the threads in the direction as typically exhibited upon prey contact.

Furthermore, we combined our empirical approach with the development of a model that was used for parametric tests to better understand the key principle behind the observed tensile behaviour. The mathematical approach is based on a modified version of the Fibre Bundle Model (FBM) [30]. To model the meta-structure characteristics of cribellar threads, we introduce polynomial stress–strain relationships and looped-like structures into the FBM. Previous theoretical studies focused on the mechanical properties of spider silks correlate the known molecular characteristics of the dragline silk of large orb weavers with their macroscopical mechanical behaviour [31–33]. Other studies were also able to treat the mechanics of well-investigated fibres to formulate multi-scale models able to explain the effect of humidity or temperature on the mechanical properties of major ampullate silks [34,35]. Compared to the fibre materials considered in these previous studies, the capture thread of *Kulkulcania* is a heterogeneous fibre bundle composed of different silk types, with its mechanical behaviour expressed through meta-structural properties. Therefore, our approach aims to explain the observed extreme elongations of cribellar spider silk only through the morphology and structure of the thread, and not the protein structure of the fibres composing it.

## 2. Material and methods

### 2.1. Spiders and sample collection

For the vertical tension experiments, *K. hibernalis* (Hentz 1842) was used from our lab breeding, *Araneus diadematus* (Clerck 1757) was caught close to Wildbach in Aachen. Both species were raised under ~21°C temperature, room humidity (~30 rh) and a 12:12 day/night cycle. Once a week food was provided, either crickets or flies. Water was provided once to twice per month by sprinkling the enclosure. Such wetted webs were not used for further research.

To measure capture threads, five webs from randomly chosen samples were used for each testing condition. The capture threads were gathered with tweezers, which were fixed to a width of 11 mm at their tips. The threads of *A. diadematus* were immediately used, as we found that storing them for 72 hours led to a loss of approximately a third of the thread samples due to dehydration. As the dry adhesive capture threads of *K. hibernalis* showed no reduction in quality during storage [36], threads were transferred to a storage sample holder until they were needed for experiments (up to 7 days).

Silk samples for microscopy and axial tensile tests were collected from the webs of adult female *K. hibernalis* living on ceilings and walls in the building of Museo Argentino de Ciencias Naturales (Buenos Aires, Argentina). Capture threads were collected on thin cardboard frames lined with double-sided adhesive tape. For a rigid fixation of the thread during the later test, it was glued along the edge of the frame window using water-based arts and crafts adhesive. For transport and storage, the loaded cardboard frames were stuck into cuts in a polymer foam material glued into a sturdy polymer box.

### 2.2. Microscopy

*Scanning electron microscop*y: Silk samples were sputter-coated with AuPd for 90–120 s and observed under high vacuum in field emission SEM Zeiss Supra (figure 1*d*), Hitachi S-4700 (figure 1*c*) or a tungsten filament SEM FEI-XL30-TMP (figure 1*e*,*f*).

**Figure 3.**
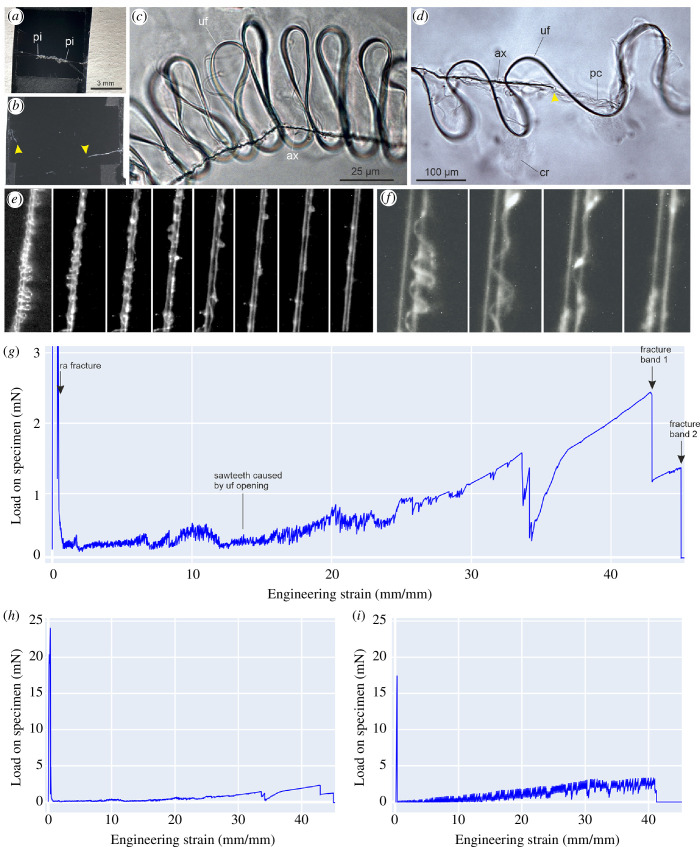
Empirical and numerical model results of the tensile mechanics of cribellar threads from *K. hibernalis*. (*a*) Test sample for which data is shown in (*e–h*) before the test. (*b*) Same sample after the test. Yellow arrowheads indicate remnants of the straightened, fractured fibre bundle left attached to the sample holder (most of the strained thread collapsed and got lost). (c) Light microscopy image of the non-strained cribellar band, showing the micro-scale (first-order) looped meta-structure. (*d*) Same as (*c*) after applying some strain. Where the uf loop has opened, the ax fibre is fractured (yellow arrowhead). (*e,f*) Series of video frames recorded on a section detail of the cribellar band during the test for which the force curve is shown in the following, showing the unravelling of the uf (first order) loops. (*g,h*) Exemplar force–elongation curve of a typical axial tensile test, with (*g*) highlighting the tensile mechanics of the cribellar bands and (*h*) showing the complete force curve including the stiff radial line breakage indicated by the initial high peak at low strain. (*i*) Force–elongation curve obtained via a model containing 3 straight elements (1 radial + 2 ax) and 8 looped meta-structures (2 uf + 6 pc). The sawtooth behaviour appears thanks to the loops unravelling. The final reached elongation is around 4000%, even though no single element in the model has a maximum elongation higher than 50%. ax, axial fibre; cr, cribellar fibres/mass; pc, paracribellar fibre; pi, piriform silk joint; uf, undulating fibre.

*Light microscopy*: Light microscopy images of the unprepared silk samples were taken with an Olympus BH-2 (figure 3*c*,*d*) or a Zeiss Discovery.V20 (figure 1*b*) on a regular glass slide.

### 2.3. Comparative vertical extension tests

Prey hitting a capture thread or trying to escape from it usually induces a load perpendicularly to the thread. To compare the performance of capture threads in such a vertical loading situation, we performed vertical tension tests. Capture threads were placed on a sample carrier (bent paper clips with two parallel wires (distance ≈8.5 mm), wrapped with double-sided adhesive tape). For the thread of *K. hibernalis*, the samples excluded the radial frame thread, and only the cribellar capture thread was transferred to the sample carrier. The integrity of all threads was controlled under the stereomicroscope (40×). The sample carrier was set onto a microbalance (JB1603/C-FACT; Mettler Toledo, Greifensee, Switzerland), which was placed underneath an arm of a motorized linear table. Both were connected to a computer to measure position, velocity and weight (as a measurement of force). An insect pin was bent into a hook, fixed to the linear table and approached the sample thread from above. This hook was used to snag the middle of the thread and then withdrawn with a speed of 1.9 mm/s. The measurement was stopped as soon as the thread ruptured and the balance was back to zero. Peak force before break was calculated by multiplying the minimal weight value by the acceleration of gravity. To measure the strain until break, the length of the thread at break (i.e. minimal weight value) was calculated by applying the Pythagoras’ theorem to the travel distance of the arm of the linear table and the halved length of the thread (4.25 mm).

The measurements were performed with capture threads of *K. hibernalis* (*n* = 33) and *A. diadematus* (*n* = 35). Constant settings with 28°C and 45% rh for the two species were chosen, ensured by using a climate chamber (HPP IPP^PLUS^, Memmert GmbH + Co. KG, Schwabach, Germany). For *K. hibernalis* and *A. diadematus,* a total number of *n* = 68 (1 setting × 2 spider species × 35/33) was measured.

Statistical analyses were carried out using R v.4.2.1 (https://www.r-project.org/). To test whether strain at break or peak force before break differed between both spider species, for each mechanical characteristic a linear mixed-effects model (LMM) was constructed with spider species as fixed factor, and spider individual as random factor.

### 2.4. Tensile testing

For longitudinal high-resolution quasi-static tensile testing, force–extension data were recorded with a T150 Universal Testing Machine (KLA, Milpitas, CA ). All tests were performed at a constant extension rate of 1% strain per second until the thread failed. This strain rate was chosen as it appears in several other works on spider silk tensile tests [2,37–39], allowing us to compare our results broadly.

For the strain calculations, we used an initial gauge length derived from the piece of the thread sample that was extended (as observed through video and post-test investigation of sample remains under the microscope). This was either the length between two piriform joints or the length between the glue fixation points at each side of the frame window. These measurements were either performed with digital callipers or the analysis of stereo microscopy images of the samples before the test, using ImageJ [40]. We think that the complex morphology of the cribellar thread cannot be simplified sufficiently to achieve a meaningful equivalent cross-sectional area to calculate stress values; thus, we chose to only record and report load forces, extensibility and strain values. Consequently, we do not provide strength and toughness values in this work. We analysed strain–force data obtained from the UTM T150 by consolidating the data into a single DataFrame using the Pandas library in Python [41].

In most of the tests, a monochrome digital video camera (Basler Ace 640 × 480 pix USB 3.0; Basler AG, Ahrensburg, Germany) was used to monitor the change in thread structure in exemplar thread segments. The camera was equipped with either a Navitar Precise Eye extension tube including a 1.33× magnification lens (Navitar, Inc., Rochester, NY) for close-ups (resulting field of view 1.3 mm × 1.0 mm at a pixel size of 2.1 µm), or with a Fujinon HF12.5HA-1B zoom lens (F1.4–F16, 12.5 mm). Videos were recorded onto the hard drive at 1 fps, using the Basler Video Recording Software.

### 2.5. Fibre bundle model

The FBM is a statistical mechanics model extensively used in understanding the behaviour of composite or complex materials under stress [30]. Originally conceived to investigate the failure of large ensembles of parallel fibres or filamentary structures under load, the model’s use has since been extended to a wide variety of materials and systems [42].

The central principle of the FBM is considering the material as an assemblage of *N* parallel fibres, each carrying a portion of the total load [30]. When an individual fibre’s threshold is exceeded, it breaks and the load it was carrying is then distributed among the remaining fibres. In its simplest form, the FBM assumes a linear elastic response from the fibres and an equal load-sharing rule, i.e. when a fibre breaks, the load it was carrying is equally distributed among the remaining intact fibres.


(2.1) F=Nkx(1−P(x))

where F is the force acting on the bundle, N is the number of fibres, k is the stiffness of each fibre, x is the elongation and $P\left(x\right)$ is the cumulative distribution function of the critical elongation distribution $p\left(x\right)$ . Equation (2.1) is already useful, despite its simplicity, especially when experiments are conducted in displacement control, e.g. via clamping the extremities of a bundle and measuring the reaction during elongation via a load cell. As shown in [30], the differential analysis of equation (2.1) provides valuable insights into the global stress–strain response of a material, helping predict phenomena like the material’s ultimate strength, strain hardening or softening, and the progressive damage leading to failure, for a variety of critical elongation distributions $P\left(x\right)$ . Further details about the implementation of a nonlinear FBM are provided in the electronic supplementary material.

#### 2.5.1. Cribellar loops

In an effort to closely emulate the mechanical behaviour of cribellate silk, we propose a ‘series of loops’ implementation (see [8] for a related problem). In this approach, each loop formed by the cribellate silk is conceptualized as a mathematical entity, characterized by a specific number of internal loops and an opening critical strain distribution $r\left(x_{c}\right)$ . When the looped structure reaches its critical strain, a loop unravels and the effective length of the fibre is increased by the length of the loop s , causing a sudden drop of the effective load and elongation on the structure. We assume a uniform opening critical strain distribution $r\left(x_{c}\right)$ . The effective displacement can be written as:


(2.2) $$x_{e}=x-\sum_{i}^{LB(x)}s_{i}$$

where LB(x) describes the subset of broken loops for that elongation value. Thus, $\sum_{i}^{LB}s_{i}$ includes all the ‘new lengths’ which were produced by each, now open, loop. The equation ruling a single series of loops is the following:


(2.3) $$F=\left\{ \begin{array}{ll} k\left(x_{e}\right)\cdot x_{e} & if,x_{e}\leq x_{c}\\ 0 & if,x_{e}>x_{c} \end{array}\right.$$

The sum given by the elongations $s_{i}$ acts as a slack, thus reducing the force acting on the fibre and allowing it to reach higher elongations. This happens while the external displacement is being applied: depending on the stochastic distribution of the critical lengths $x_{c}$ and the slacks s , different series of increasing and suddenly decreasing measured forces can be observed. This generates a sawtooth curve, where the slope of each tooth is given by the stiffness of the silk forming the loop thread, k . We also introduce the concept of hierarchical series of loops. We imagine a looped fibre S1 ; at each S1 loop opening corresponds a fracture of a different fibre S2 , adherent to S1 . The stresses continue to be distributed between the fibres as before. Thus, the effective elongation of fibre S2 depends on the internal loop opening, plus the openings already occurred in S1 , as S2 is also not under tension until the elongation unfolds completely the loop opened in S1 .


(2.4) $$x_{e_{S2}}=x_{S2}-\sum_{i}^{LS2}s_{i_{S2}}-\sum_{i}^{LS1}s_{i_{S1}}\cdot C_{s}$$

Here $C_{s}$ is the coefficient that regulates how much of the strain coming from S1 is being transmitted to S2 . The load on S2 can be obtained by inserting (2.4) into (2.3). Through iterative computations, we can model the unfolding of loops of different dimensions and with different mechanical properties. It is important to notice that this description follows the same structure of the FBM: when studying a bundle of fibre B and a series of loops L being pulled in parallel, the force measured in the overall system will be $F_{tot}=F_{B}+F_{L}$ . Further considerations are discussed in the electronic supplementary material.

## 3. Results and discussion

### 3.1. Morphological analysis of the cribellar capture threads in *K. hibernalis*

The meta-structure of the cribellar capture threads of *K. hibernalis* is illustrated in figures 1 and 2 has been partially described previously [12,13,21]. Here, we highlight the unique hierarchical loops-on-loops pattern of the thread (figure 2, table 1).

**Table 1.** Summary of the hierarchical loop meta-structure of the *Kukulcania* cribellar thread. Note that for the numerical study, only the first-order looping was considered.

level	description	looped element(s)	straight element
first order (micro-scale)	basic element of brushed-up fibres deposited along the straight axial line	uf, pc, cr	ax
second order (meso-scale)	regular undulating of the cribellar band held together by framing fibre	ax	ff
third order (macro-scale)	coiling of the whole cribellar thread (consisting of two cribellar bands) around the radial line	ff	ra

*Macro-structure (third-order looping)*: The paired cribellar band is attached to a stiff radial carrier line (ra) with piriform (pi) silk joints applied at regular intervals. The pi joints seem to envelop the complete cribellar thread and represent fixed points beyond which cribellar thread extension rarely occurs. The thread exhibits an irregularly coiled structure on the macro-scale and mostly runs along the ra line.

*Meso-structure (second-order looping)*: The cribellar capture thread is composed of two cribellar bands, each of which exhibits a meso-scale looping structure slightly twisting regularly in a patterned way, following the shape of a sinusoidal wave. This macro-structure is seemingly stabilized, at least during the construction phase, by a thin and fragile auxiliary fibre (ff; in [22] called ‘axial fibre’) attached to the outer surface of cribellar band loops (figure 1*b*). This fibre, however, could not be found in many samples investigated in the light and SEM and, where present, likely plays no significant role in the tensile mechanics, which is why it was not considered in the theoretical approach.

*Micro-structure (first-order looping)*: Each of the two cribellar bands contains a single axial fibre (ax; called ‘supporting fibre’ in [22]), a single major undulating fibre (uf, of minor ampullate silk), three thin paracribellar fibres (pc), forming loops alongside the major undulating fibre and the cribellar nanofibre mat (cr) forming regular puffs (in light microscopy images and videos barely visible). The diameters of the fibres were reported in [22] as uf 2.0 ± 0.2 μm, pc 0.4 ± 0.1 μm and ax 0.4 ± 0.1 μm. The cr mat does not envelop completely the other three structures, but instead surrounds and links the fibre loops like an adhesive tape. The uf and pc fibres form a continuous series of loops along the entire length of the ax fibre. The major uf exhibits a highly regular loop structure and a flattened cross-section, which increases its contact surface with the other structures in the thread.

### 3.2. Evidence of extreme elongation of *Kukulcania* cribellar threads

**Figure 4. F4:**
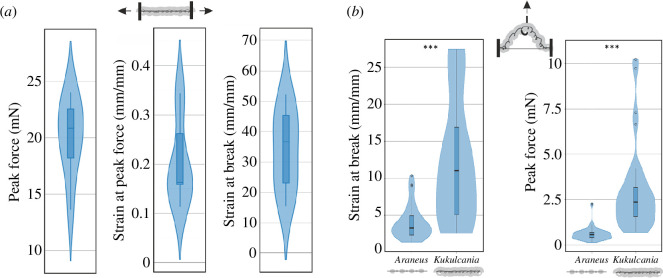
Summary of data from extension tests with cribellar (*K. hibernalis*) and ecribellar (*A. diadematus*) capture threads. (*a*) Results from longitudinal (axial) tensile tests of *K. hibernalis* threads. (*b*) Results from vertical tension tests of *K. hibernalis* and *A. diadematus* capture threads.

Exemplary force–elongation curves for the capture thread of *K. hibernalis* are shown in figure 3*g*,*h* [Fig F4] (for all force curves see the electronic supplementary material). In total, eight empirical tests were conducted and analysed. Averages and standard deviations of forces and elongations are shown in table 2 and figure 4*a*.

**Table 2.** Means and standard deviations of the radial line peak force, the peak force reached by the cribellar band after the failure of the radial line, the strain value at peak force (i.e. radial breaking) and the strain value at the total failure.

test	species	radial line peak force (mN)	peak force before break (mN)	strain at peak force	strain at break
longitudinal	*K. hibernalis*	20.11 ± 3.39	1.08 ± 0.54	0.19 ± 0.09	33.7 ± 12.9
*vertical*	*K. hibernalis*		2.95 ± 2.36		12.2 ± 8.3
	*A. diadematus*		0.59 ± 0.37		4.0 ± 2.2

For the vertical extension (i.e. for *K. hibernalis* pulling at a cribellar thread segment of defined length between piriform joints perpendicularly to the ra line, excluding the ra line, figure 4*b*), we found a significant superior mechanical behaviour compared with the viscid capture threads of *A. diadematus* (tables 2 and 3). Strain at break was increased by more than three times and peak force before break even by five times leading to higher extensibility and tensile force in the cribellar capture thread (figure 4*b*). Peak forces in this test were lower than in the axial tension test because the ra line was not loaded. Ultimate strain was lower probably because the vertical extension test induces more stress concentrations than the axial tension test leading to premature breakage.

**Table 3.** Statistical differences of mechanical characteristics of spider capture threads, measured at vertical extension. Strain at break and peak force before break were measured with cribellate capture threads of *K. hibernalis* and with ecribellate capture threads of *A. diadematus*. For the results reported below, an LMM was constructed with spider species as fixed factor, and spider individuals as random factor.

mechanic characteristic	fixed effect	d.f.	*χ* ^2^	*p*
strain at break	spider species	1	13.733	<0.001
peak force before break	spider species	1	33.257	<0.001

After elongation, the ax fibre was found broken into several parts; however, these segments were held together in place, probably by adhering to the pc fibres and the cribellar mat (figure 3*d*).

### 3.3. Tensile behaviour of *Kukulcania* capture threads

In the quasi-static tensile tests, we observed a series of peaks of various intensities. The first peak, visible in figure 3, is caused by the radial thread fracture between two piriform anchorages; this was confirmed by the videos captured during the elongation experiment. After the fracture of the radial thread, we expected an interval in the force–elongation curve at zero load, given by the free rotation of the cribellar bands, which have to realign themselves in order to feel any tension. However, we always observe a reaction from the silk. This hints to a mechanism, we think actuated by the cr mat, to easily propagate stresses in the bands. Videos captured during the empirical tests revealed that the looped meta-structure is still intact after radial line breakage and is unravelled stepwise along a narrow segment. Thus, the following part of the force–elongation curve consists of the irreversible opening and unravelling of the cribellar bands. In particular, the peaks and drops of lower intensity are indicators of the unravelling of the loop meta-structure in the uf and pc (figure 3 *e*,*f*). The unravelling seemed to occur in both cribellar bands more or less simultaneously. The ax fibre appeared to fracture repeatedly and/or slip-stick during loop openings of the undulating fibres. The multiple fracture of the ax fibre has been reported before for the cribellar threads of *Psechrus* spiders [19]. Ongoing load bearing of the ax line, even after failure was assumed to be possible through its interaction with the undulating fibre mediated by the adhesive cribellar mass. This was interpreted as a crucial mechanism to prevent catastrophic failure or sudden thread slack.

We were unable to clearly determine from the videos the possible effects caused by the opening of the pc fibres. At high strain, when no fibre loops were visible any longer in the video, the straightened fibre bundle kept on extending considerably. At this stage, potentially pc and cr fibres might still exhibit a slack and continue to be straightened out, further delaying the complete failure of the bundle. Complete failure seemed to occur when the uf fibres and the cribellar mat failed in any point of the overall length. Here, the two halves of the cribellar thread (and accordingly each of the two straightened undulating fibres) usually fractured independently, resulting in two major force drops towards the end.

Our experiments show *Kukulcania* capture threads can reach an elongation of up to 4500%. To our knowledge, this puts the *Kukulcania* threads as the most extensible silk-based materials found in nature. Previous studies about spider silks and other natural silks stop at 1100% for simple threads [20,43] and 2500% for other cribellar threads [21]. The values found in our study compete with artificial materials, as polymer-based gels, which store their hidden lengths at the molecular level thanks to the chemical and physical cross-linked polymer networks inside of them: hydrogels reach high extensibilities of 1000–1500% [44,45], while an organogel capable of reaching strain values of 10 500% was developed by Zhang *et al*. [46]. Another solution for high stretchability is provided by some metal foils, which can be stretched up to 43 000% [47]. However, the processing of these materials differed substantially from the one by *Kukulcania*. We think that, by transferring the dry fibre-based meta-structural principle of *Kukulcania* threads, engineers may design new fabrics with outstanding extensibility while using known polymer materials. Such fabrics may be highly useful in single-use tools which benefit from high elongations, especially in dynamic situations, e.g. safety devices, parachutes and medical devices.

### 3.4. Parameters of the FBM

We defined a mathematical model of the cribellar thread based on 11 elastic elements in parallel. Here, we only considered the first-order loop structure, which, according to the empirical observations, seems to have the greatest effect on prolonged extensibility of the fibre bundle. Six series of loops are identified as *paracribellar fibres*. Two series of loops are named *undulating fibres*. Two linear elements, called *axial components*, have a diameter five times lower than the undulating fibres, and are adherent with all the series of loops included in the model. The last element, identified as the *radial component*, does not interact with the other elements. We imagine that all the elements composing the cribellar bands share their stresses thanks to two mechanisms: the presence of adhesive mechanisms between them, and the cribellar mat surrounding the fibres. Following the method illustrated in the previous section and in the electronic supplementary material, we thus consider the ax fibres as elements capable of breaking or slipping multiple times while still contributing to the mechanical response of the bands. We were unable to collect and test single ax fibres from *K. hibernalis*. Therefore, for this work, we suppose the stress–strain curve of such fibre is similar to pseudoflagelliform silk supposedly causing the initial slope in tensile tests of the cribellar capture threads of uloborid and deinopid spiders measured in [48]. We represent this curve with a cubic law, showing a stiffening phase towards the end of its elongation. The maximal strain is assumed as 0.50±0.05 . We consider that the mathematical representation of the axial component in our model is the combination of several factors, all taken into account in its equation: the cribellar mat’s mechanical response to strain, the axial component of the undulating fibres (i.e. the sectors of undulating fibres which may completely straighten up after some local openings), and the mechanical response of the ax fibre itself. We thus chose to represent the force acting on the axial component with equation (2.3), while the elongation is given by equation (2.4). The transmission coefficient $C_{s}$ was chosen after conducting a series of parametric tests, of which a summary can be found in the electronic supplementary material. The additional hidden lengths propagated to the axial component are coming from the loops appearing in the undulating fibre. The force acting on the undulating fibre is represented by the looped fibre equation (2.3). The elongation of the undulating fibre is given by equation (2.2). Grannemann [22] showed that these meta-structures are produced by the minor ampullate glands. We thus consider the undulating fibre being stiffer than the ax fibre, as inferred from the data available on minor ampullate silk on other species in the literature [2]. We have no information on the mechanical properties of the pc fibres. Therefore, we chose to use the same laws and equations as for the ax fibres. Our SEM images revealed that loop size is similar for pc and uf fibres, while the diameter of these fibres differed. We transferred these characteristics to the model. For simplicity, and due to the lack of experimental single fibre measurements, we assume the ra, uf and pc fibres as linearly elastic and with a similar Young’s modulus. We obtain the force–elongation behaviour of the cribellar bundle by increasing in a discrete fashion the elongation, and numerically solving the system of 11 coupled equations generated by the 11 parallel elements.

### 3.5. Key factors affecting tensile mechanics of looped fibre meta-structures

We explored the potential behaviours of the cribellar thread structure by conducting a collection of analytical and parametric analyses using our model. Full results are shown in the electronic supplementary material. The analysis shows that the hidden length provided by the undulating fibres alone is not sufficient to justify the extreme elongations appearing in the capture threads. This result was obtained taking into consideration the size and number of loops appearing in our sample. This suggests that the pc fibres and the cribellar mat provide further elongations to the overall structure. Parametric results showed that it is not sufficient to define a looped meta-structure to achieve the extreme properties reached by the silk products of *K. hibernalis*. It also revealed that the distribution of the critical strains at which the loops in the pc and uf fibres open themselves could have a huge role in the growing forces observed towards the end of the elongation of the cribellar bands. Uniformly distributed critical strains, decoupled from the loop sizes, provide a positive stiffness to the overall structure. Further notable features are: (i) the regions of the force–elongation plot where the overall stress is zero could be generated by the opening of numerous loops in a small time interval, due to their similar critical force; and (ii) a trivial but important consequence of having more than one looped meta-structure is the creation of less patterned sawteeth. A single looped fibre, even if characterized by loops with a uniform distribution of their critical strain along a huge range of values, will only have one loop opening appearing at a time, since each loop opening causes a strain gain which resets the force across that structure.

### 3.6. Discussion of the results of the theoretical study

Results of the numerical model are shown in figure 3*i*. For simplicity, we assume that the radial component is constituted by only one element. The most elongating uf transmits its generated displacements s to the ax component with a transmitting coefficient $C_{s}=0.999$ . The uf and pc fibres are assumed to have the identical dimensions and material properties for the sake of simplicity. The parameters used are shown in table 4. We assumed that the critical strain at which each loop opens is a statistically distributed variable going from 0% to 300% of the size of the loop. The radial element breaks at low strain values, leading to an early, high peak, followed by a series of sawtooth created by the continuous generation of slacks given in equation (2.4). The increasing force values seen towards the end of the force–elongation curve are given by the polynomial stress–strain law chosen for the axial component, $\sigma =0.1\cdot \varepsilon +5\cdot \varepsilon^{3}$ . Notice that no component of the model (e.g. single silk fibre material) has a maximum elongation higher than 50%; yet, a maximal strain of 4000% of the meta-structure was observed. Compared to the theoretically obtained curve, the empirical result shown in figure 3*g*,*h* shows a bigger variance in the amplitude of the peaks creating the sawtooth-like behaviour; we think this may be caused by slip-stick effects occurring during ax fibre fracture and undulating fibre loop openings. It can also be noticed that the empirical sawtooth peaks have, on average, a smaller amplitude; we could not replicate this behaviour without using unrealistic numbers for the geometrical parameters (such as relative fibre diameters), i.e. numbers that strongly differ from the microscopically observed structure. Other considerations about differences between the empirical and theoretical behaviours are found in the electronic supplementary material. We want to clarify here that some model parameters used for the shown solution have been arbitrarily chosen (within realistic ranges), as due to the adhesive nature of the cribellar mat the empirical quantification of single element properties is difficult, if not impossible, to accomplish. Different theoretical solutions, reaching even higher ultimate strains, could be found by modifying the parameters defining the cribellar bundle, increasing, for example, the number of loops per millimetre for each fibre, or by representing the cribellar mat with a 12th equation containing additional hidden length. Many properties of the silk fibres forming the *Kukulcania* cribellar threads empirically studied in this work are unknown as, for example, the mechanical properties of the ax, uf and pc fibres; the mechanical properties of the cribellar mat (with exceptions, see R [15]); the adhesive properties of the fibres and the cribellar mat to each other, the effect of the spider leg movement during the spinning onto fibre properties, the topology of most fibres, and the consequences of the dynamics of the spigots on the mechanical properties of single fibre materials. However, we think that this model achieves a good fit of the maximal elongation and sawtooth-like behaviour of the experimental data and provides a relatively simple explanation of the extreme extensibility of this peculiar cribellar thread, without the need to introduce materials with extreme properties in the model. This result could explain the properties of several capture threads produced by different cribellate spiders, and not only *K. hibernalis*, which also present high ultimate strains while presenting a simpler structure [12]. Furthermore, the model is a starting point for exploring how composite meta-structures of looped and straight fibres can be tailored to maximize tensile strain and toughness, and to design new materials for applications requiring extreme deformations.

**Table 4.** Parameters used for the numerical solution presented in figure 3*i*.

name	number of fibres	number of loops	relative diameter	Young’s modulus (GPa)	critical strain	loop critical strain	loop relative length	$C_{s}$
radial component	1	/	1	7	0.2	/	/	/
axial component	2	/	0.2	0.1	0.5	/	/	0.999, uf
uf	2	200	0.175	7	/	0.150 ± 0.145	0.038 ± 0.002	/
pc	6	200	0.035	7	/	0.150 ± 0.145	0.038 ± 0.002	/

## 4. Conclusions

In this study, we quantitatively characterized the mechanical properties of the capture threads produced by the Southern house spider (*K. hibernalis*). The investigation revealed that the threads exhibit a distinctive tensile behaviour attributed to their hierarchical looped microstructure adjoining straight lines, which contributes hidden length by controlled unravelling and significantly delays catastrophic fracture of the fibre bundle. Vertical and axial tension tests, supported by microscopy and mathematical modelling, revealed that this material has the most outstanding extensibility of any known natural silk fibre material. The gradual uncoiling of the structure and the sequential breaking of linear fibres are key mechanisms that delay failure under tension. The model presented in this work helps the understanding of the extreme properties found in *Kukulcania* capture threads, and could also be applied to other cribellar spiders. Furthermore, these findings not only contribute to the understanding of spider silk mechanics but also have implications for the design of advanced materials with extreme extensibility with potential applications in safety equipment and medical devices. Future research could focus on the translation of these biological insights into practical engineering solutions.

## Data Availability

The data of this work is reported in the tables and in the electronic supplementary material [49].
